# Supplementary material for: Are carbon emissions trading and green financial instruments synergistic? -Comprehensive quantitative research based on content analysis
Source: PLoS One. 2024 Mar 7;19(3):e0298601. doi: 10.1371/journal.pone.0298601 (PMC10919601; doi:10.1371/journal.pone.0298601)
Supplement: S1 Data — (ZIP) [file pone.0298601.s001.zip › Data/地级市Z_F工作报告.zip!/║╙▒▒/│╨╡┬╩╨/└┤╫╘╣1⁄2╓┌║┼í╛┬φ┐╦╩2╛▌═°í┐_2017.docx]

　各位代表：

　　现在，我代表市人民政府，向大会报告工作，请予审议，并请市政协委员和列席会议的同志提出意见。

　　一、本届政府工作回顾

　　市第十三届人大一次会议以来，在省委、省政府和中共承德市委坚强领导下，全市上下认真学习贯彻党的十八大和十八届三中、四中、五中、六中全会精神，深入落实习近平总书记系列重要讲话和对承德作出的“定位于建设京津冀水源涵养功能区，同步考虑解决京津周边贫困问题”重要指示，紧紧围绕“三区一城”发展定位，积极践行新发展理念，主动适应引领经济新常态，抢抓机遇，攻坚克难，砥砺奋进，全市经济社会发展取得显著成绩，为全面建成小康社会，建设生态强市、魅力承德奠定了坚实基础。

　　综合经济实力稳步提升。2016 年生产总值达到1432.9亿元，年均增长7.4%；全社会固定资产投资完成1632.8亿元，年均增长12.4%；规上工业实现增加值471.7亿元，年均增长7.1%；全部财政收入完成147 亿元，其中公共财政预算收入82.1 亿元；社会消费品零售总额达到543亿元，年均增长11.6%。县域经济规模持续扩大，8个县的经济总量全部超过百亿元，宽城县突破200亿元。经济发展规模和水平跃上新台阶。

　　结构调整取得明显成效。三次产业结构由2012 年的15.7：52.9：31.4 调整为16.6：45.6：37.8。 实施亿元以上传统产业技改项目344 个，净增规上工业企业107 家。绿色产业蓬勃发展，文化旅游、钒钛新材料、新型建材等十大产业增加值占GDP 比重达到35%，高新技术产业增加值年均增长10.7%。清洁能源电力装机达到380 万千瓦，被列为全国新能源示范市。旅游总收入达到506亿元，增长2.1 倍。电子商务交易和网络零售额达到550 亿元，滦平、围场等3 个县成为全国电子商务进农村综合示范县,承德县被评为省级服务外包呼叫中心基地。新增国家和省级现代农业示范园区12 个，市级以上农业产业化龙头企业404 家，农业产业化经营率达到68.7%，跻身全国六个休闲农业与乡村旅游示范市行列。

　　协同发展和对外开放迈出坚实步伐。抢抓京津冀协同发展重大机遇，120个重大事项列入国家及京津冀战略规划，实施对接合作项目734个，完成投资1048 亿元。携手中关村打造协同创新共同体示范区，大数据电子信息、生命医学大健康、节能环保三个产业园相继落地。承德高新区升格为国家级高新区，新增省级高新区2 家。打通国道111 线、津兴线等6 条连接京津的瓶颈路，环首都3 县5 条城际公交直连北京。与京津之间交通基础设施、产业、生态等多方面合作日益深化。全方位多渠道对外开放，外贸出口总额15.7 亿元、年均增长32.8%，实际利用外资5.5 亿美元、年均增长11.3%，引进市外资金1981亿元。承德海关开关运行。隆化保税仓库建成投用。协同发展的叠加效应不断显现。

　　生态环境质量保持华北地区领先。造林绿化386万亩，森林覆盖率达到56.7%。出境断面和饮用水源地水质达标率达到100%，水环境质量保持全省第一。深入开展大气污染防治，实施治理项目908 个，取缔燃煤锅炉923台，推广使用高效节能炉具13.3万台。投放纯电动公交车385 台，黄标车实现“清零”目标。超额完成省下达的主要污染物减排任务，空气优良天数保持在270 天以上。推动建立滦河流域生态补偿机制，在全国率先开展跨区域碳汇交易，成功争列国家生态文明先行示范区和全国水生态文明建设试点市，生态引力持续增强。

　　基础设施建设实现重大突破。承张、承赤高速建成通车，新增高速公路382 公里、达到758公里；新建改建国省干线428 公里、农村公路5078 公里。承德机场即将通航，平泉机场开通国内首条军民合用短途运输航线。张唐铁路竣工通车，新增铁路运营里程215 公里、达到795 公里。新建输变电站18 座、通讯基站6476 个。双峰寺水库具备蓄水条件，丰宁抽水蓄能电站二期开工，南郊热电即将投入运营。一批重大基础性工程的建设使长期制约发展的短板得到明显改善，对助推经济社会发展起到至关重要作用。

　　新型城镇化进程加快。有序放开城镇落户限制，全面推开居住证制度。全市常住人口城镇化率达到49%，提高7.1 个百分点。中心城市功能持续改善，实施水电路气等市政基础设施项目668 个，建成区面积从97平方公里扩大到117平方公里。县城承载力进一步提升，宽城“产教城”一体化模式在全省推广。特色小城镇加快发展，建制镇达到93 个，全国重点镇达到20 个。争列和建设兴隆燕山峡谷等省级美丽乡村精品片区5 个、重点村878 个，农村人居环境不断改善。

　　重点领域改革扎实推进。大力推进“放管服” 改革，非行政许可事项全部取消，审批事项减少148 项，向县区下放行政权力260 项，“三级平台”“两个代办”实现全覆盖。全面推行商事登记制度改革，市场主体达到18.14万户、注册资本2690亿元，分别是2012年的1.8倍和3倍。加大国有资本市场化运营力度，组建国控集团并实现健康发展。金融业获得长足进步，发起设立全国首支农业产业发展引导基金，新增外埠银行4家、保险证券机构10 家，上市挂牌企业达到29家。农村综合改革稳步推进，土地确权登记颁证595万亩，流转率达到27.8%，市县农村产权交易平台全部建成。供销社综合改革、国有林场改革走在全国前列。

　　各项社会事业全面进步。民生投入累计达到927 亿元，占全部财政支出的83.8%。城乡居民人均可支配收入分别达到24856 元和8736 元，年均增长7.4%和12%。脱贫攻坚成效显著，63.08万贫困群众稳定脱贫，506 个贫困村摘帽。新增城镇就业18.3 万人，农村劳动力转移就业16.3 万人，高校毕业生就业率达到90.9%，城镇登记失业率控制在4.5%以内。城乡居民医疗保险、养老保险参保率分别达到95%和99%。改造棚户区住房3.33 万套、农村危房10.62 万户。义务教育均衡发展，职业教育全省领先。县级公立医院改革实现全覆盖，医药卫生体制改革走在全省前列。城乡公共文化服务体系不断完善，营子区被评为全省公共文化服务示范区。双拥共建结出新硕果，荣获全国双拥模范城“八连冠”。安全生产、食品药品安全形势保持平稳，法治承德、平安承德建设扎实推进，双桥区、双滦区被列为全国和谐社区建设示范城区。文化体育、人口计生、民族宗教、外事侨务、人民防空、史志档案、统计调查、气象地震、防灾减灾、对口援疆、邮政通信、妇女儿童、老龄、残疾人等工作取得新成绩。

　　政府自身建设不断加强。深入开展党的群众路线教育实践活动、“三严三实”专题教育和“两学一做”学习教育，坚决贯彻中央“八项规定”精神，认真落实全面从严治党责任，加大行政监察和审计监督力度，推进行政权力依法公开透明运行，政府系统作风建设发生显著变化。完善科学民主决策机制，建立重大决策专家咨询、公众听证、合法性审查制度，依法行政能力进一步增强。自觉接受人大法律监督与工作监督、政协民主监督以及社会监督、舆论监督，共办理人大代表建议738 件、政协提案971 件，按时办复率均为100%。

　　各位代表！刚刚过去的2016 年，面对经济持续下行的巨大压力，面对稳增长、调结构、转方式的艰巨任务，面对精准脱贫和生态建设的重大责任，我们保持沉着冷静、稳中求进的战略定力，强化打基础利长远、久久为功的责任担当，发扬拼搏实干、奋发作为的进取精神，推动经济发展呈现稳中向好、动力增强的新态势，实现了“十三五”良好开局。一年来，我们在直面困难挑战中精准发力，一批稳增长的政策措施落地见效。围绕推进供给侧结构性改革，出台了一系列务实管用的政策措施，“三去一降一补”取得阶段性成效。提前完成钢铁、煤炭去产能年度目标任务。深入开展企业帮扶年和环境优化年活动，投放银企帮扶资金190 亿元，为企业减负13 亿元，帮助120 家企业正常生产。重点项目建设三年攻坚行动成效明显，实施亿元以上重点项目841个、完成投资1124 亿元。营商环境进一步改善，市场主体和注册资本分别增长14%和39.7%，经济下滑势头得到有效遏制。一年来，我们在绿色发展中加快动能转换，一批新兴产业呈现强劲发展势头。着力培育壮大十大绿色产业，引进德鸣大数据产业园、南开大学大数据研究院等大数据电子信息项目30 个，总投资685 亿元。启动实施天然矿泉水、山泉水开发项目21 个，农夫山泉、汇源等6 家饮品名企落户承德，全市总产能达到320 万吨。引进战略投资者实施天宝矿业集成技改，力促传统产业优胜劣汰、提档升级。承德冰雪季激活冬季旅游，“大槐树”成为全省农村电商三大平台之一。被列为第二批国家全域旅游示范区创建城市、京津冀大数据综合试验区和国家物流标准化试点市。十大绿色产业增加值占GDP 比重超过“两黑”产业10 个百分点，新动能正在撑起发展新天地。一年来，我们在改善生态环境中巩固放大优势，一批重大环境治理与修复工程深入实施。把建设京津冀水源涵养功能区，进一步改善生态环境作为重大政治责任和发展责任，成功争列山水林田湖综合整治试点和国家绿色矿业发展示范区，“6743 工程”和“双百行动”成效显著。实施总投资73.6 亿元的滦河武烈河生态治理工程，全面开展潘家口水库网箱养鱼清理。探索编制《自然资源资产负债表》，并在全国地级市中第一个公开发布。环境质量继续保持京津冀前列。一年来，我们在积极主动融入京津中发展自己，一批制约发展的瓶颈实现突破。全面推进京冀津冀战略合作协议落实，7 个贫困县与京津建立结对帮扶工作体系，与天津签署“1+4”战略合作协议，与北京朝阳医院等5 家知名医疗机构深度合作。京承津承交通一体化加快推进，承平高速控制性工程开工建设，京沈高铁承德段全面推进，承津城际铁路启动前期。着力提升协同发展的承载能力，“一带三区”城市战略全面启动。实施开发区改革发展三年攻坚行动，省级开发区整合至10 家，新建省级以上科技创新服务平台29 个，园区承载力和科技支撑能力显著增强。一年来，我们直面问题多措并举，脱贫攻坚和民生改善实现重大进展。188 个贫困村出列、12.8 万贫困群众实现稳定脱贫，“政银企户保”金融扶贫经验在全国推广，平泉食用菌扶贫模式被评为“全国十大产业扶贫范例”。年初政府工作报告中向全市人民承诺的20 件惠民实事全部完成。提高特困人群医疗救助水平试点顺利推进，房地产领域遗留问题、国有企业改制职工安置、老城区学校幼儿园建设等一批累积多年的“老大难”问题得到有效解决，踏踏实实为人民群众办了一批好事实事。

　　各位代表!我市经济社会发展取得的成就，得益于党中央、国务院的亲切关怀，得益于省委、省政府和市委的坚强领导，得益于市人大、市政协和社会各界的大力支持，得益于全市广大干部群众的团结奋斗、艰苦努力。在此，我代表市人民政府，向全市383 万人民，向人大代表、政协委员，向各民主党派、工商联、无党派人士和人民团体，向驻承人民解放军、武警部队官兵和政法干警，向一直以来关心和支持承德经济社会发展的国内外各界朋友，表示崇高的敬意和衷心的感谢！

　　各位代表！在看到来之不易成绩的同时，我们也时刻保持着清醒的头脑，我们深知在全市经济社会发展中还存在不少困难和问题。主要表现在：经济总量小、产业层级低、创新能力弱、新兴产业发育慢，结构性矛盾突出，调结构、转方式极为迫切；住房保障、教育医疗、交通出行、城市管理、食品药品和公共安全等方面的短板依然突出；贫困面大，贫困人口多，贫困程度深，脱贫攻坚任务十分艰巨；部分行业企业生产经营困难，财源支撑能力弱，“三保”支出压力大，财政收支矛盾突出，财政金融风险上升；少数干部懒政怠政、不担当、不作为，一些改革举措和政策迟迟落不了地，营商环境亟待改善，等等。对这些矛盾和问题，我们不能回避，也不能绕着走，而要以改革创新的理念思路，以攻坚克难的勇气决心，以咬定青山不放松的恒心毅力，把压力挺起来，把责任扛起来，把担子担起来，全力以赴做好政府工作，绝不辜负各位代表和全市人民的信任与重托!

　　二、今后五年发展目标和主要任务

　　未来五年，是全面建成小康社会的决胜期，也是承德大有作为、大步前进、后来居上的关键期。我们面临千载难逢的重大发展机遇。纵观大势，国家京津冀协同发展全面深化，扶贫攻坚集中发力， 供给侧结构性改革持续推进，我省配套跟进“一揽子”政策措施，为我们激发企业活力、扩大有效供给、促进动能转换带来重大利好。立足承德，我市地理区位优越、历史文化厚重、生态环境良好、各类资源丰富的优势日益凸显，再加上立体综合交通网络的形成，这些其他地区不可比拟的条件所产生的独特魅力，正在转化为对资本、项目、人才的强大吸引力。一大批绿色产业项目相继落地，并将陆续进入收获期，为未来承德发展积蓄了宝贵的后劲，为我们后来居上实现跨越发展创造了条件。更为宝贵的是全市政通人和万事兴的良好局面已经形成。市第十四次党代会紧跟时代步伐，描绘了承德未来五年发展的宏伟蓝图，激发了广大干部群众干事创业的激情和斗志。只要我们抓住机遇、凝神聚力，只要我们用好政策、乘势而上，只要我们坚定信心、发愤图强，就一定能谱写出实现中华民族伟大复兴第一个百年梦想光辉灿烂的承德篇章！

　　按照市第十四次党代会的总体部署，今后五年政府工作的指导思想是：全面贯彻党的十八大和即将召开的十九大精神，坚持以邓小平理论、“三个代表”重要思想和科学发展观为指导，深入学习贯彻习近平总书记系列重要讲话精神，统筹推进“五位一体”总体布局，协调推进“四个全面”战略布局，认真践行新发展理念，适应把握引领经济发展新常态，大力推进供给侧结构性改革，坚定不移走加快转型、绿色发展、跨越提升新路，全面落实“三区一城”定位和“12345”总体目标思路，加强预期引导，强化创新驱动，全力稳增长、促改革、调结构、惠民生、防风险，确保如期实现脱贫摘帽、全面小康，为建设生态强市、魅力承德而不懈努力。

　　今后五年发展的主要预期目标是：生产总值达到2160 亿元，年均增长7.5%左右；规模以上工业增加值年均增长7.5%以上；固定资产投资年均增长10%以上；全部财政收入和公共财政预算收入力争年均增长10%以上；城乡居民人均可支配收入年均增长9.5%以上，城镇化率达到55%以上。重点完成好以下七大任务：

　　（一）坚定不移加快转型升级，构建绿色发展新格局。坚持“差异化、特色化、增量调整”方向，加快发展十大绿色产业，力争绿色产业增加值占生产总值比重超过60%，构建起多元化、中高端的现代产业体系。

　　推动传统产业迈向中高端。完成压减炼铁462万吨、粗钢563 万吨去产能任务的同时，强力推进矿山钢铁企业整合技改、绿色发展，实现“产能压减、效益翻番”。强力推进国家绿色钒钛产业基地建设，研发高端钒钛钢、钒钛制品、全钒液流电池、钛材等高端钒钛产品，钒钛新材料产值达到1300亿元。强力推进装备制造业智能化发展，培育壮大军民融合产业，汽车零部件、输送装备、智能仪器仪表传统产业迈向工业4.0，加快发展通用飞机、新能源汽车等先进装备制造业，产值达到1000 亿元。强力推进新型建材产业产品研发、链式配套、集成服务，打造京津冀新型建材及建筑产业化基地，新型建材产值达到700 亿元以上。强力提升食品医药产业，引进和培育一批龙头企业，建设中国北方最大的道地中药材种植加工基地和全国知名的医药产业聚集区，绿色食品及生物医药产业产值达到1200 亿元以上。

　　实现战略性新兴产业挑大梁。坚持优先用力，推动大数据、矿泉水、节能环保、清洁能源等优势新兴产业率先发展，构建支撑全市产业和财源的新“四梁八柱”。围绕建设“京津冀大数据综合试验区”和“国家绿色大数据中心”，着力打造“五大基地”，形成大数据存储、应用、云计算及软件研发、相关硬件制造等全产业链条，构建大智移云中高端产业集群。围绕建设“中国高锶矿泉城”，唱响“京津水源·皇家矿泉”金字招牌，积极引进名企名牌嫁接改造本土企业，培育产值超20 亿元企业3家，天然矿泉水、山泉水产量达到600 万吨以上。围绕建设“环保技术国际合作基地”，加快承德高新区节能环保产业园和县区特色产业园区建设，形成“核心园+特色园区”的“1+N”产业架构，节能环保产业产值达到1100 亿元以上。围绕建设国家级清洁能源产业基地，推广风力发电、光伏发电新技术应用，打造风光储一体化产业格局，清洁能源电力装机达到1400 万千瓦，发电量突破300 亿千瓦时。

　　壮大现代服务业生力军。推进旅游产业高端化、智慧化、国际化、全域化发展，集全市之力把2018 年第三届全省旅发大会办成精彩纷呈的盛典，把180 公里的坝上公路打造成享誉全国的最美风景大道。大力完善旅游基础设施，提高旅游服务标准，培育航空体验、房车营地、VRAR 等高端旅游业态，建设生命医学大健康和国际养老养生休闲基地，扩大我市旅游康养产业国际品牌知名度和影响力。抢抓北京冬奥会机遇，大力发展冰雪运动，建成金山岭、六里坪、大黑山、崔梨沟等一批具有国际水准的滑雪度假小镇和以冰雪体验为主的5A 级景区。完善冰上运动设施，培养输送一流的冰上运动人才，把避暑山庄冰球队打造成为有国际影响力的品牌球队。以建设国家全域旅游示范区为总抓手，努力实现产业全域联动、景区全域优化、服务全域配套目标，加快一季游向四季游、观光游向休闲游、景区景点游向全域游转变，实现从山庄外庙时代向全域旅游时代的新跨越。深入实施文化强市战略，大力发展文化创意、影视演艺、数字传媒等文化产业，“21 世纪避暑山庄”争创国家级文化产业示范园区，进入文化旅游综合收入千亿城市行列。大力发展金融服务、交通运输、建筑劳务、研发设计等生产性服务业，积极发展医养结合、娱乐健身、家政服务、便民商业等生活性服务业，建成国家公共检验检测认证服务平台。加快省级物流园区建设，推进电子商务、跨境电商发展，打造京津冀辽蒙区域重要物流枢纽城市。到2021 年，以文化旅游康养为引领的现代服务业增加值占GDP比重达到50%以上。

　　做优做强县域经济。坚持差异化、特色化发展，依托各县资源禀赋，大力发展特色主导产业，力争经济总量超200 亿元的县、全部财政收入超20 亿元的县分别达到3 个以上。大力推动产教城融合发展，每县建成一个产业特色鲜明、教育配套到位、宜居宜业宜养的产教城园区。建成2 万人以上中心镇16个。支持平泉等有条件的县撤县建市。大力深化“放管服”改革，为各县竞相发展、活力迸发创造良好环境。

　　（二）坚定不移打赢脱贫攻坚硬仗，力促农业农村发展呈现新局面。坚持精准扶贫、精准脱贫基本方略，在全面完成脱贫攻坚目标任务的同时，将全市农业农村发展提高到一个新的水平。

　　决战决胜脱贫攻坚。突出精准要求，因人因户因村施策，扶贫方式向“绣花式”“滴灌式”转变。突出产业扶贫，实施功能农业扶贫、光伏扶贫、旅游扶贫、生态扶贫等专项行动，培育壮大九大特色扶贫主导产业，让每一个贫困户、每一个贫困群众都有增收项目、都有脱贫门路。突出重点区域，加大对深度贫困县支持力度，改善深山区、革命老区、民族地区生产生活条件，易地扶贫搬迁基本完成。突出重点人群，通过实施医疗救助、社保兜底等办法，让特困群体和“三无户”生活无忧。突出扶贫模式创新，推广“三零”“一地生四金”“一林生四财”等扶贫模式，“政银企户保”实现全覆盖，确保贫困人口如期全部脱贫，建档立卡贫困村全部出列，贫困县全部摘帽，与全国全省同步全面建成小康社会。

　　加快发展现代农业。以农业供给侧改革为主线，生态农业、美丽农业、品质农业和功能农业取得长足发展，创意农业、智慧农业不断壮大，高端优质农业供给能力显著提升。加快“1＋2＋3＋N”现代农业园区建设，建立京承、津承农产品共建共享互认体系，市级以上农业产业化龙头企业达到500 家，“三品一标”农产品占比65%以上，全市现代农业产值超1000 亿元，建成全国首个功能农业扶贫示范区，力争国家农业高新技术产业开发区创建成功。

　　建设美丽文明乡村。坚持脱贫攻坚、现代农业、乡村旅游、民俗文化、山区综合开发与美丽乡村建设“六位一体”推进，按照“五美”要求，开展好“四改、四化、两治、四提”14 个专项行动，打造围场皇家猎苑、隆化热河皇家温泉等精品片区20 个以上，基本实现美丽乡村建设全覆盖，让广袤的农村大地富起来、美起来、强起来。

　　（三）坚定不移加强生态文明建设，巩固提升区域竞争新优势。倍加珍惜生态优势，改善提升生态水平，实现生态环境区域最优、全国领先，把生态环境打造成为承德发展核心竞争力。

　　持续增强生态屏障支撑能力。围绕“两河、六路、四区”布局，深入实施京津风沙源治理、京冀水源林、再造“三个塞罕坝”等重大生态工程，加快“四大林业功能区”建设，新增造林绿化385 万亩，有林地面积达到3600 万亩，活立木蓄积量超过1亿立方米，森林覆盖率达到60%以上。加快全流域水环境综合治理和生态修复，双峰寺水库建成投用，每县至少开工建设1 座中小型水库，新增蓄水4 亿立方米，水资源控制率达到20%以上，湿地面积保持在6 万公顷以上，水土流失治理率达到55%以上。实行最严格的水资源管理制度，全面落实“河长制”，确保出境断面和饮用水源地水质达标率保持100%，水环境质量华北领先。

　　坚持不懈开展污染减排治理。全面完成国家绿色矿业发展示范区创建“6743”工程，骨干矿山企业全部达到省级以上绿色矿山标准。打好蓝天保卫战，加强京津冀区域联防联控，深入开展散煤综合治理，推动集中供暖和清洁能源供暖，加大电代煤、气代煤和洁净型煤推广力度，清洁能源供热面积达到300 万平方米。中心城区公交车全部使用清洁能源替代，空气质量优良天数比例达到85%以上，PM2.5 平均浓度值达到国家二级标准，大气质量在全国74 个重点城市中的位次明显提升。严控工业固废排放和农村面源污染，加快建立农业清洁生产技术体系，保障农产品质量和人居环境安全。

　　构建生态文明法律制度体系。划定生态保护红线，实行最严格的生态保护制度，探索用能权、用水权、排污权、碳排放权等市场化有效途径，打造生态产品交易平台。推动建立潮河流域跨界水环境横向生态补偿机制。积极推动生态环境保护地方立法，深化自然资源资产负债表综合应用，实现自然资源资产台账全覆盖。全面开展自然资源责任审计，建立差异化绩效考核机制，构建较为完善的生态文明法律制度体系，努力让承德天更蓝、地更绿、水更清，积累更多的生态财富，保护好可持续的根基命脉，擦亮发展的“金字招牌”。

　　（四）坚定不移促进协同发展，提升对外开放新境界。京津冀协同发展和“一带一路”战略机遇难得，必须牢牢抓住、用足用好，把眼光放远、把格局做大，全面提升对外开放的层级和水平。

　　加快交通基础设施一体化建设。加快铁路建设，确保京沈客专2019 年通车运行，力争承津城际铁路建成通车，力促北京城郊铁路向承德延伸，建成“一环九射”铁路网，融入“轨道上的京津冀”。加快航空发展，有序开通承德机场至全国各大区域航线和重点国际航线，年客运吞吐量达到100 万人次以上。加快7 个通用机场建设，基本建成“一运八通”航空网，融入京津冀世界级机场群。加快公路建设，承平、承克高速建成通车，承津、赤曹高速开工建设，高速公路里程达到900 公里以上，建成“一环八射”高速公路网，实现县县通高速；彻底打通与京津的瓶颈路，建设干线公路1000 公里、农村公路5000公里，所有建制镇和80%以上的乡通达二级以上公路，所有行政村路面宽度达到5 米以上。实现京津冀交通一卡通，推进城乡客运一体化，基本形成京津承“一小时交通圈”。

　　加快产业一体化发展。积极承接北京非首都功能疏解，有效利用天津辐射带动作用，着力打造承德与京津优势互补、错位联动的一体化发展产业生态。强化对接合作，促进承德资源优势、生态优势与京津科技优势、人才优势相结合，以十大绿色产业为主体，形成京津研发-承德制造、京津消费-承德生产服务的产业联动发展新格局。加大京津总部经济引进力度，力争一批大企业、大集团在承德设立区域性总部或分支机构。用好IPO 免排队优惠政策，吸引京津企业落地承德，促进承德传统优势企业与京津优质企业嫁接合作，借助京津外力，实现产业层级和水平大幅提升。

　　加快优质要素一体化共享。加快打造环京津卫星城和“微中心”，吸引京津优质要素向承德聚集。创新教育、医疗、文化等领域合作模式和机制，推动与京津之间人才、科技、产品等生产要素自由流动，共享共建，带动我市公共服务水平快速提升和相关产业加速发展。加快农产品检验检测、新型建材质量标准、就业任职等资格互认，农产品交易、产权交易、生态产品交易等市场互通，实现协同发展成果的最大化。

　　加快全方位对内对外开放。适应并跟进南资北移大趋势，扩大与长三角、珠三角和港澳台地区合作，建设发达地区产业北上重要承接地。借力国家“一带一路”建设，全力拓展东亚、欧洲和俄罗斯市场。探索“内陆港”“保税区”等外向型经济模式，支持有条件的园区设立保税监管场所，形成对外开放功能区，培育区域经济新高地。力争到2021 年利用外资达到4 亿美元，进出口完成8 亿美元，对外开放结出更加丰硕成果。

　　（五）坚定不移深化改革创新，着力培育经济发展新动能。牢固树立改革意识和创新思维，坚持以改革增动力、添活力，以创新促发展、求突破。

　　全面推进重点领域改革。深化简政放权、加强监管、优化服务，促进行政许可权相对集中使用。深化投融资改革，新增多层次资本市场上市企业40 家，金融业增加值年均增长15%以上，对经济增长贡献率达到20%，力争把我市打造成为京津冀一体化东北翼区域金融中心。深化国企国资改革，完善国控集团等国有企业法人治理结构和国有资本运营机制，大力发展混合所有制经济。扎实推进农业农村综合改革，完善农村土地“三权分置”办法，发展多种形式农业适度规模经营，推进农村集体产权制度改革，形成农民持续增收的新机制。加快推进城市综合执法改革，建立城市管理工作标准规范体系。培养优秀企业家队伍，构建新型政商关系，激发非公有制经济活力，新增市场主体10 万户，民营经济占比达到75%以上。

　　全面推进科技创新改革。创新科技投入机制，力争R.D 投入占GDP 比重达到全省平均水平。鼓励社会资本投身科技研发和科技成果转化主战场，形成政府主导、企业主体、社会参与的科技创新体系。以承德·中关村协同创新共同体示范区为引领，构建“政产学研企”创新体系，大幅提升企业创新能力、产业创新能力和区域创新能力。到2021年，建成国家级钒钛新材料工程技术研究中心等一批区域性协同创新平台，科技孵化和创新园区达到50 家，各类科技研发和公共服务机构超过200 家。全市科技型中小企业达到2000 家以上，高新技术企业达到100 家，高新技术产业增加值占规上工业比重超过25%。科技进步贡献率达到60%以上。

　　全面推进开发区体制机制改革。深入实施开发区改革发展三年攻坚行动，改革开发区机构设置、人员编制、薪酬分配和选人用人制度，创新招商机制、投融资体制和服务模式，加快“九通一平”和“新九通一平”配套建设，形成特色优势产业集群，力争承德高新区进入国家级“第二方阵”，双滦经开区建成国家级经济技术开发区。全市开发区生产总值年均增长20%以上、占比达到50%，财政收入年均增长10%以上、占比达到45%以上，真正成为改革开放的先行区、创新驱动的示范区、经济发展的增长极。

　　（六）坚定不移推进新型城镇化，迈出城乡统筹发展新步伐。积极融入京津冀世界级城市群，以建设国际旅游城市为引领，实现“五城联创”目标，构建起“以城带乡、城乡一体”的现代城镇体系。

　　中心城市辐射带动力显著提升。加快实施“一带三区”城市发展战略，拓展城市空间，中心城区建成区面积达到130 平方公里、人口达到100 万以上。下大力推进老城区“中疏”和“治堵”，力争城市组团间骨干通道不少于2 条，形成中心城区“八纵八横”骨干通道,提高城市道路通行能力；首条城市轨道交通建成运营，城市公共交通取得重大突破。挖掘城市文化内涵，调整优化业态，严控建设标准，落实“多规合一”。持续开展城市环境综合整治，加强精细化、精致化、精美化管理，着力提升城市品质，打造智慧城市应用示范城市。抓好南北新城起步区、引导区和重点区建设，加快实施临空经济产业区、国际滑雪度假区、皇家康养休闲区等投资超千亿的基础设施和产业项目，形成新城组团基本框架。

　　县城小城镇规模品质显著提升。强力推进县城建设攻坚行动，深入挖掘不同地域文化，促进产教城融合发展，打造一批精品街区和地标性建筑，提高政府空间管控水平和综合治理能力。加快构建“一核、三带、多点”市域城镇空间布局，以滦平县、双滦区、双桥区、高新区、隆化县、围场县为重点的御路文化城镇带，以丰宁县、滦平县、兴隆县、承德县和营子区为重点的环京津城镇带，以平泉县、宽城县为重点的出海城镇带初步形成。加快承德县与中心城区同城化步伐，推进营子区资源枯竭型城市转型发展。积极推进撤乡设镇，建制镇占乡镇总数比例接近或达到全省平均水平。

　　城乡基础设施水平显著提升。加快水源地和供水管网建设，完善城市河道干支流防洪及水景观体系，保证防洪和供水安全。强化海绵城市建设理念，提高城市排水防涝减灾能力。实施“宽带承德”工程，统筹推进骨干通讯网络建设。加快城乡电网及各类老旧管网改造升级，加强地下综合管廊、煤气、天然气、充电桩、污水和垃圾处理、“两场一厕”等公共设施建设，进一步完善城市功能。通过几年坚持不懈的努力，让我们的城市不仅“面子”更好看，“里子”也越来越厚实。

　　（七）坚定不移保障和改善民生，更好满足人民群众新期待。牢固树立以人民为中心的发展思想，持续加大民生投入，促进城乡居民收入稳定增长，不断提高民生保障和服务能力，努力让发展更有温度，幸福更有质感。

　　大力促进就业增收。全面落实更加积极的就业政策，实现就业困难群体帮扶全覆盖。千方百计提高各阶层收入水平，多渠道增加农民收入，提高企业职工和机关事业单位职工收入，确保城乡居民收入和增长幅度达到或超过全省平均水平。

　　织密民生保障网。实施全民参保登记计划，基本养老保险覆盖全体职工和适龄城乡居民，基本医疗保险覆盖全民，失业、工伤、生育保险覆盖所有法定群体，护理保险全面实施。不断提高“两线合一”标准，建立低保标准自然增长机制，城乡低保标准年均增长10%以上，达到全省平均水平，特困供养服务机构集中供养能力达到60%。加强保障性安居工程建设，基本完成中心城区棚户区改造任务。

　　办好公平优质教育。优化基础教育布局，促进义务教育均衡发展，构建现代职业教育体系，注重高等教育内涵发展，教育发展主要指标达到全省先进水平。中德应用技术大学承德分校建成并招生，新建改扩建中小学150 所，新增公办幼儿园80所。

　　提升医疗康养水平。实施中医药强市战略，深化医疗卫生协同发展，全面推进公立医院综合改革，完善三级医疗服务网络，建立覆盖城乡居民的基本医疗卫生制度。加强计划生育服务管理，扎实落实全面两孩政策。完成城市社区20 分钟养老服务设施布局，每千名老年人拥有床位40 张以上，实现城市社区综合服务设施全覆盖，力争农村社区综合服务设施覆盖率达到50%。

　　发展文化体育事业。推进现代公共文化服务体系建设，实施文化惠民和文明素质提升工程，传承弘扬优秀传统文化，深化德育教育，创造诚实守信的社会环境。加快发展竞技体育，培养高水平运动人才。大力开展全民健身运动，构建全民健身设施网络，打造城市社区15 分钟健身圈，全市各类体育场地达到4500 个以上，每个社区、每个村都建成一处公共体育健身场所。

　　深入开展安全创建。严格落实安全生产责任制，进一步做好食品药品安全、公共安全、森林草原防火和防汛抗灾等工作，建立完善立体化信息化社会治安防控体系，有效防范化解各类风险，确保社会大局和谐稳定、人民群众安居乐业。

　　各位代表！全面建成小康社会的伟大任务历史地落在了我们这一代人肩上。只要我们万众一心、不怕困难，信念坚定、矢志不移，就一定能够走出暂时的困难、打拼出一片新天地，就一定能够把宏伟蓝图变成美好现实，就一定能够铸就承德发展新的辉煌！

　　三、2017年重点工作

　　今年是全面落实市第十四次党代会精神的第一年，也是新一届政府的开局之年，做好今年各项工作对我们挑战很大、考验很大。我们一定要认真贯彻习近平总书记系列重要讲话精神和治国理政的新理念新思想新战略，全面落实党中央国务院、省委省政府和市委各项决策部署，扑下身子抓落实，撸起袖子加油干，以经济社会发展的优异成绩迎接党的十九大胜利召开。

　　今年全市经济社会发展主要预期目标是：生产总值增长7%，规模以上工业增长5%以上，固定资产投资增长10%，全部财政收入和公共财政预算收入均增长5%，实际利用外资增长10%以上，出口总额增长5%以上，社会消费品零售总额增长10%以上，城乡居民人均可支配收入分别增长8%和10%以上，城镇登记失业率控制在4.5%以内，万元生产总值能耗下降4%左右，常住人口城镇化率达到50.5%，地表水水质优良率保持在95%以上，PM2.5 年均浓度及化学需氧量、二氧化硫、氨氮、氮氧化物减排完成省下达目标。重点做好八个方面工作：

　　（一）大力推进经济持续稳定增长。把稳增长作为首要任务，千方百计稳定提升传统产业，以更大的决心培育新的经济增长点。

　　全力实施项目攻坚。把项目建设作为重中之重，深入实施项目攻坚三年行动和“33591”工程，确保完成固定资产投资1776 亿元以上。建立落实市县领导“五个一”包保机制和“七人小组”工作机制，抓好推介招商、集中开工和观摩拉练，全年新开工荣盛新能源产业园等亿元以上项目500 个，福田汽车高精尖零部件、河北航研制冷设备、磬棰湾国际商业街等225 个重大产业和服务业项目投产运营，努力形成开工项目热火朝天、竣工项目接二连三、后备项目源源不断的生动局面。

　　加快发展实体经济。制定出台支持实体经济发展实施意见，设立总规模100 亿元发展基金，对市场前景好、发展潜力大的重点企业给予重点扶持。筛选100 家以上重点企业，实行市县两级领导一对一包保、点对点帮扶。进一步放宽投资准入，力争民间投资增长18%以上，民营经济增加值增长6%以上。持续实施以创业创新带动就业为重点的“双创”行动，推动“个转企、小升规、规改股、股上市”，新增市场主体2.2 万户、规上工业企业50 家以上，使小企业铺天盖地，大企业顶天立地，市场活力和社会创造力竞相迸发。

　　全面促进消费升级。深入开展“大健康、新医疗”行动，开拓医养结合、健康管理、康复护理、体育健身等新兴服务消费。深入开展增品种、提品质、创品牌专项行动，争创中国驰名商标和省著名商标10 件以上，省级名牌优质产品25 项以上。深入开展“互联网+流通”行动，实施电子商务进社区进农村工程，推动线上线下融合发展，电子商务交易和网络零售额达到670 亿元。加强消费者权益保护，营造良好的市场秩序和消费环境。

　　（二）大力推进供给侧结构性改革。以提高质量和效益为核心，进一步优化产业结构，加快培育发展新动能，力争十大绿色产业增加值占生产总值比重提高到36%以上。

　　深入推进三去一降一补。坚定不移去产能，压减煤炭90 万吨、生铁104 万吨、粗钢190 万吨，稳妥做好职工安置和债务处置工作。加强房地产市场调控，强化价格管控和引导，规范房地产市场秩序，促进房地产市场平稳健康发展。强化地方政府债务和金融管理，确保不发生系统性和区域性风险。落实减少涉企收费政策，深化政银企对接合作，切实减轻企业负担，提升企业效益。

　　全力办好旅游业发展大会。坚持国际视野规划、一流标准建设,突出生态、扶贫、特色、全域、全时，紧锣密鼓做好第三届全省旅发大会各项筹备工作，高水平办好首届全市旅发大会。力争“国家一号风景大道”主场馆和重大节点工程主体基本完工，沿线交通基础设施改造，美丽乡村和高端民俗、农家乐建设初具规模，游客中心、旅游公厕、驿站、旅游标识等配套服务设施同步推进。集中开展两个旅发大会沿线、重点片区“两改一拆”和旅游环境改造提升工程，为大会成功召开和全市旅游发展创造优良环境。

　　加快培育壮大新兴产业。深入实施绿色产业三年倍增计划。谋划成立旅游发展投资公司和文化产业发展投资集团，建立旅游产业发展基金，加快双桥大稿国际艺术区、围场御道河谷文化旅游区等重大文化旅游产业项目建设，力争文化旅游业实现增加值580 亿元、增长10%以上。中经承园、斐讯大数据产业园等10 个项目开工建设，华明服务外包产业园、智慧承德云平台等15 个项目投入运营，德鸣大数据特色小镇建设初具规模，大数据产业体系基本形成。中国（承德）高锶康养矿泉水研究院、院士工作站挂牌成立，矿泉水博物馆建成投用，新增矿泉水产能200 万吨以上。加强与中国宜兴环保科技园战略合作，启动节能环保产业园“五个一”建设。围绕集成装配式建筑产业化，配套整合、扩大市场，新增新型建材企业10 家以上，实现产值180亿元。支持承钢公司、天大钒业、锦科科技、建龙特钢等一批钒钛新材料企业做大做强，启动矿业整合重组和传统产业技改升级攻坚行动，实施超亿元技改项目80 个以上。

　　持续提升农业有效供给。紧紧盯住京津两大消费市场，深入实施农产品加工业倍增和品牌培育计划，中能昊龙滦平冀康产业园、隆化10 万头肉牛深加工等一批项目开工，森源食用菌等20 个农产品精深加工项目投产达效，农业产业化经营率达到69.7%。完善提升14 个省级农业产业化联合体，培育建成8-10 个富硒农产品示范试验区，加快打造京津菜篮子绿色有机产品供应基地。办好首届京津冀中药材发展大会，建设燕山中药材经济核心示范区。进一步优化农业种养结构，再调减籽粒玉米30 万亩，新增高端设施蔬菜4 万亩，食用菌达到14.6 亿袋，马铃薯播种面积达到90 万亩，中药材栽培达到77 万亩。加强新型职业农民培养，培育新型农业经营主体和服务主体1500 个以上。

　　（三）大力推进精准扶贫精准脱贫。坚持精准扶贫、精准脱贫基本方略，真抓实干，吹糠见米，确保14.6 万农村贫困人口稳定脱贫，力争平泉县、兴隆县摘帽。

　　夯实基层基础。坚持问题导向，科学制定贫困人口脱贫和贫困县摘帽计划，开展脱贫攻坚“四看”活动。规范“四证一册”，做到一户一人不落发放到位。建立建档立卡动态管理机制、部门间数据比对和信息共享机制，实现贫困人口有进有出、动态管理，提高脱贫攻坚精准度。

　　聚焦重点区域。按照跨县连片统筹发展的思路，打破区域空间布局，以丰宁、围场、隆化3 个深度贫困县为重点，充分利用中央和省扶持政策，把资金、项目、资源集中倾斜，实行资源整合、权益捆绑、统筹推进，放大整体效益，打造政府、企业、市场、农户多方共赢的扶贫产业聚集区。

　　狠抓精准落地。以产业扶贫为主攻方向，加快打造“五个百万基地”“六条扶贫产业带”，壮大九大特色扶贫主导产业，培育旅游扶贫试点村120个。实施“百村万户阳光扶贫工程”，建成村级光伏扶贫电站130 个以上，新增户用分布式光伏1 万户。实现智慧电商扶贫服务站全覆盖，力争每个贫困村至少发展1 个脱贫产业，每个贫困户至少参与一项稳定增收项目。抓好45 处集中安置小区“交钥匙”工程，新启动搬迁70889 人，确保贫困群众搬得出、稳得住、能致富。加大教育、医疗救助和社会保障扶贫力度，抓好“提高贫困人口医疗保障救助水平解决因病致贫返贫问题”省级试点，落实“三免一助”和农村义务教育学生营养改善计划，把该保的保好、该兜的兜住。

　　创新攻坚机制。充分发挥151 个省直、190 个市直和563 个县区直驻村工作队的定点帮扶作用，积极引导企业开展爱心扶贫，形成全社会帮扶的新机制新格局。制定完善资产收益与股份合作利益联结办法，培育壮大贫困村集体经济，构建稳定脱贫长效机制；统筹整合财政涉农资金，持续加大扶贫投入，强化资金和项目监管；切实落实脱贫攻坚责任制，实施最严格的评估考核，确保脱贫得到群众认可，经得起历史检验。

　　（四）大力推进以京津为重点的对外开放。坚持以京津冀协同发展为引领，全方位扩大开放，借外力，增内力，激活力。

　　在精准对接京津上求突破。紧盯纳入京津冀协同发展规划重大事项，推动北京帮扶事项和天津“1+4+5”合作协议落地见效。加快津冀六沟产业园区和北京（承德）影视产业基地建设，中德应用技术大学承德分校主体基本完工。加快营子怡达首都农产品应急储备基地、高新区农产品冷链物流产业园等5 个大型综合市场建设，提高区域承接和服务能力。加大IPO 政策宣传，研究出台相应配套政策，吸引京津一批优质企业落户承德、挂牌上市，新增上市企业6 家以上。

　　在补齐发展短板上求突破。承德机场开通石家庄、上海、广州等5 条航线，平泉机场开通京津等周边城市航线。承平高速全面开工，承克、承津高速公路和承津城际铁路加快推进，新建续建干线公路520 公里，建成农村公路600 公里。大力提升园区基础设施建设和配套功能，所有开发区规划区全部实现“七通一平”和免费WiFi全覆盖，承德高新区、双滦经开区规划区完成“九通一平”，力争每个园区新入驻京津企业10 家以上。大力实施科技创新“四大工程”，新培育认定高新技术企业10 家、科技小巨人企业10 家、科技型中小企业200家以上。

　　在扩大开放合作上求突破。抓住国内外大企业大集团在京津冀抢滩布局机遇，组织好百家央企京企进承德、百家津企进承德、中国承德国际经洽会等重大招商推介活动，开展好“一带三区”以及水源地承德绿色产品北京行、天津行等专题招商，组织团队赴长三角、珠三角地区开展重点招商，强化以商招商、产业链招商，提高招商活动的精准性和有效性，全年引进市外资金500 亿元以上。加快河北鹏涛跨境电子商务平台和保税仓库建设，新增出口备案企业20 家，外贸进出口增速位居全省前列。

　　（五）大力推进生态环境治理与修复。以国家生态文明试验区创建为抓手，深入开展“蓝天、碧水、净土、雷霆”四大行动，调高标尺，加大力度，进一步提升环境质量。

　　出重拳治理大气污染。以冬季清洁取暖、工业污染防治、机动车尾气治理、扬尘综合整治、城乡增绿扩容为重点，严格落实“1+18”大气污染综合治理各项措施，加快电代煤、气代煤等清洁能源替代工程，全面取缔城市和县城建成区35 蒸吨及以下燃煤锅炉，升级改造工业锅炉222 台，治理施工工地面积1000 万平方米，中心城区清洁能源公交车达到300 台。扎实推进“三个一百”专项行动，完成复垦披绿100 家、取缔关闭100 家、绿色改造矿山企业100 家，压减矿权140 个，减少矿区面积20平方公里，打造全国矿山环境治理新亮点。确保PM2.5 年均浓度下降2.5%以上，优良天数达到276天以上。

　　下大力提升水环境质量。深入贯彻国家“水10条”和省“水50 条”，落实好市县乡村四级“河长制”，滦河武烈河综合治理开工20 公里以上。全面完成潘家口水库网箱养鱼清理任务。推动落实滦河流域横向补偿政策，争取建立潮河流域生态补偿机制。

　　多举措净化土壤环境。开展土壤污染调查、风险评估、治理修复，抓好畜禽养殖废弃物处理和资源化利用、危险废物治理、涉重金属企业清理整顿、关停搬迁企业污染场地治理修复等专项整治，加强农业面源污染防治，实现化肥、农药零增长。推进山水林田湖综合治理，完成造林绿化60 万亩，新发展经济林28 万亩，治理水土流失面积500 平方公里，建成国家森林城市，实现“五城联创”首创成功。

　　建机制压实监管责任。严格落实环境保护“党政同责、一岗双责、失职问责、终身追责”工作机制，加强联合执法、区域执法、交叉执法，严厉打击各类环境违法行为。健全市县乡村四级网格化监管体系，完成环保机构监测监察执法垂直管理制度改革。探索建立领导干部自然资源资产离任审计制度，打造全国自然资源资产负债表编制应用的样板和示范。

　　（六）大力推进重点领域和关键环节改革。坚持上下联动、协同发力，更好发挥改革牵引作用，更大释放改革红利。

　　以优化营商环境为重点深化“放管服”改革。深入开展行政审批规范化标准化建设，优化再造政务审批流程，实现市县区行政审批局全覆盖。开展“减证便民”专项行动，全面清理各种证明和手续。探索实施“互联网+政务服务”，整合各部门信息资源，打破信息“孤岛”，让干部和数据多跑腿，让群众和市场主体少跑腿、不跑腿，享受最大程度便利。

　　以绩效管理和破解投融资瓶颈为重点深化财税金融改革。加快财政事权和支出责任划分，强化绩效预算管理，大力压减一般性支出，把每一笔钱都花出效益，用在刀刃上。完善市本级和各县区投融资平台运行机制，建立健全资本注入、风险控制、项目决策制度，与多层次资本市场全面对接，有效扩大融资渠道。积极引进外埠金融机构，新设县级银行支行5 家、村镇银行3 家。搭建银企项目合作精准对接平台，力争金融业增加值增长13%以上。

　　以保障农民权益为重点深化农村综合改革。围绕“三权分置”改革，放活承包土地经营权，有序推进农业适度规模经营。稳步推进农村集体产权制度改革，抓好双滦区农村集体经济股份合作制改革国家试点，认定市级农村股份合作制经济组织80 家以上。以平泉、围场两县为试点，稳步推进农业水价综合改革。组建承德农林科学院。推动供销合作社综合改革，所有乡镇全部建成新型基层供销社。完善御道口牧场管理体制，完成32 家国有林场改革任务。

　　以保障群众健康权益为重点深化医药卫生改革。深入开展县级公立医院综合改革示范县创建、8 家城市公立医院综合改革，全面实行药品零差率销售。优质医疗资源向农村和城市社区倾斜，推进分级诊疗制度实施，健全完善基本医疗保险、大病保险和医疗救助等救助体系，加强计划生育全程服务管理改革，落实好全面两孩政策。

　　以提高资产资本运营效益为重点深化国企国资改革。积极推进国有企业分离办社会职能工作，全面启动33 家企业“三供一业”分离移交。完善国控集团运行机制，健全国有资产监管体制，推进市属国有企业统一监管全覆盖。建立绩效考核、资本运作、薪酬分配制度，探索完善国有资本有效运营机制，确保国有资产保值增值，把人民的共同财富切实守护好、发展好。

　　以提高绩效水平为重点深化开发区体制机制改革。落实省委、省政府关于开发区改革各项政策要求，积极引入市场机制，深化开发区机构编制、人事薪酬、行政审批、投融资平台建设等重点领域改革，破解影响开发区发展的体制机制障碍和瓶颈制约，推动开发区建设提速提档、增效进位。

　　（七）大力推进新型城镇化。坚持以人为本，加快构建产教城融合、城乡一体的发展格局，全市常住人口城镇化率提高1.5 个百分点。

　　实施“一带三区”城市发展战略。探索城市建设运营新机制，全面推进与中信产业基金、华夏幸福、北京新奥、北京供销社等的战略合作，谋划对接重点产业和基础设施项目50 个以上，确保新开工28 个、总投资318 亿元，加速形成优质产业和优势资本聚集效应。滦河经济带完成道路建设36.1公里，启动10 公里地下综合管廊建设，推动“一带三区”各板块之间良性互动、协调互补、错位发展，打造城市经济新的增长极。

　　实施县城建设攻坚行动。兴隆、平泉和承德县基本建成产教城园区，实现8 县产教城园区全覆盖。扎实开展智慧城市建设，县级数字化城管平台全面建成。创新城市执法管理体制机制，市县区实现城市管理领域机构综合设置。因地制宜推进丰宁汤河温泉小镇、平泉契丹文化小镇等22个特色小镇建设，稳步实施“村改居”和户籍制度改革，支持农业转移人口市民化。

　　加快美丽乡村扩面提质。加强历史文化名村、民族民俗、传统村落、历史建筑保护开发，坚持“六位一体”统筹推进，抓好5 个省级、12 个市级精品片区、50 个旅游示范精品村建设，高标准完成特色小镇和省级重点村建设任务，进一步改善农村人居环境，建设一批田园风光、传统文化与现代文明兼具的美丽乡村。

　　（八）大力推进惠民利民“办实事”工程。民生是为政之要，我们一定时刻放在心头、抓在手上。通过向社会公开征集和反复认真研究，决定今年集中力量实施“十大民生工程”，再办20 件与人民群众切身利益息息相关的实事。一是就业创业工程。①千方百计稳定和扩大就业，城镇新增就业4万人以上，下岗失业人员再就业1.5 万人，农村劳动力新增转移就业3.8 万人，扶持大学生、返乡农民工、复转军人、下岗职工创业4000 人以上，让更多的人在劳动中创造财富，在奋斗中实现人生价值。二是教育提升工程。②进一步改善办学条件，改造农村义务教育学校50 所，中心城区新建改扩建世纪城学校、冯营子初中等11 所中小学和幼儿园，启动市特殊教育学校迁建工程，让所有的孩子成就梦想，使更多的家庭实现希望。三是医疗健康工程。③所有公立医疗机构全部取消药品加成，药品价格和大型医疗设备检查费用分别下降15%和10%以上，进一步减轻百姓就医负担，让广大群众看得起病、看得好病。④城乡居民养老保险参保率保持在95%以上，基础养老金标准提高到每人每月90 元，失业人员保险金提高到月人均1010 元。⑤将农村建档立卡贫困户、特困供养人员等六类贫困人口全部纳入医疗保障救助扶贫实施范围，合规医疗费用报销比例达到86%以上,解决好因病致贫返贫问题，使困难群众心里有温暖，生活有奔头。四是破产企业职工安置工程。⑥积极筹措资金，基本完成国资、建安、商贸系统31家破产国企职工安置工作，使上万名职工生活得到有效保障。五是老旧小区改造提升工程。⑦完成老城区华富苑小区、油坊小区等16 个老旧住宅小区的污水管网、路面硬化、楼顶防水、外墙保温等改造。启动老旧小区加装电梯试点工作。⑧加强老旧小区物业管理，重点推进老城区中兴路小区、双柳小区等30 个老旧小区物业管理试点，逐步提升老旧小区物业管理水平。六是回迁房建设和棚户区危房改造工程。⑨下大力解决城市建设领域遗留问题，中心城区新建回迁房9.5 万平方米，完成安置回迁48.1 万平方米、货币化安置和商品房回购安置23.2 万平方米。⑩新改造棚户区6587 套，农村危房1.6 万户，让更多住房困难家庭告别棚户区，让广大人民群众在住有所居中创造新生活。七是中心城区“畅洁绿亮美”工程。对中心城区拥堵问题实行综合整治，启动总长7.4 公里的牛圈子沟至闫营子南北新通道建设。对34 处路口、267 处信号灯进行升级改造。新增电动公交车100 辆，新开通罗汉堂至水泉沟、双滦区医院至锦绣城枢纽站等4 条公交线路，调整优化13 路公交车线路，增加11 路公交车密度，延长28 路公交车运营时间。新建一批智能停车场和立体车库，新增停车位2500 个；健全完善停车场管理办法，严格停车场收费、服务管理。实施广电路、竹林寺等30 条街巷和小区街道照明工程，照亮百姓身边路，让群众出行更方便、生活更舒心。八是智慧城市和中心城区管网建设工程。中心城区、县城和A 级以上景区实现免费WIFI 全覆盖，加快推进乡镇4G 通信网络、行政村光纤全覆盖。中心城区建成便民网上缴费平台，力争实现水、电、供热、燃气等各类缴费网上结算。新建和改造中心城区供热管网50公里，燃气、自来水、污水、雨水管线各20 公里以上。九是全民健身工程。武烈河复式河床步行系统全线贯通，增加亲水设施8 处。为120 个村配备体育健身设施，新建完善一批体育场馆、运动公园、健身步道，为广大群众休闲健身提供良好环境。十是“厕所革命”工程。新建改建旅游公厕520 座，其中中心城区新建和改造公厕42 座，全部达到国家A 级标准。同时，我们还将全力以赴抓好安全生产、食品药品安全、森林草原防火等各项工作，严厉打击违法犯罪，有效防控各种风险，确保社会大局和谐稳定。

　　民生无小事，枝叶总关情。我们一定时刻把群众的安危冷暖放在心上，把人民的嘱托期待记在心头，把该办能办的事情竭力办好，坚决兑现向全市人民作出的庄严承诺！

　　四、切实加强政府自身建设

　　面对决战决胜全面小康的光荣使命和艰巨任务，各级政府必须进一步增强落实新发展理念的行动自觉，进一步坚定新常态下推动发展的信心决心，进一步提升走好新路的能力水平，恪尽职守、永不懈怠，在改革创新、依法履职、勤政务实、担当负责、廉洁高效政府建设上迈出更坚实的步伐。

　　（一）坚持从严治政，落实全面从严治党要求。牢固树立和自觉践行政治意识、大局意识、核心意识、看齐意识，严守政治纪律和政治规矩，更加自觉地在思想上政治上行动上同以习近平同志为核心的党中央保持高度一致，确保政令畅通、令行禁止。严格执行《准则》和《条例》，加强和规范党内政治生活，强化党内监督。深入推进党风廉政建设和反腐败工作，切实履行好“一岗双责”，加大行政监察和审计监督力度，严肃查处侵害群众利益的不正之风和腐败问题，打造一支“绝对忠诚、干事担当、干净自律、充满活力”的公务员队伍。

　　（二）坚持依法行政，切实维护社会公平正义。深入贯彻全面依法治国要求，崇尚法治、敬畏法律、依法办事。坚持科学决策、民主决策、依法决策，推进市县综合执法改革，严格公正文明执法。坚持政府常务会议学法制度，推动法律顾问制度市县区全覆盖，提高依法行政能力和水平。依法接受人大及其常委会的法律监督，自觉接受人民政协的民主监督，主动接受社会和舆论监督。加大政务公开和信息公开力度，让政府决策和执行全过程晒在阳光下，置于监督中。

　　（三）坚持为民施政，全面提升行政服务效能。始终把人民群众是不是赞成和拥护作为检验政府工作的“定盘星”“度量衡”，深入基层、调查研究、问计于民，对群众和市场主体反映强烈、要求迫切的问题积极创造条件，认真加以解决，定下来就办、办就办好。全面落实“双随机、一公开”监管机制，把该放的放开，该管的管好，让政府瘦身、给市场松绑、为群众谋利，让幸福在老百姓家门口升级。

　　（四）坚持实干实政，驰而不息改进工作作风。认真落实中央八项规定精神，坚决防止“四风”反弹。大力弘扬“塞罕坝”精神，扎实开展“转作风、优环境、促发展”活动，对标先进，调高标尺，事争一流。健全正向激励和容错纠错机制，给干事者鼓劲，为担当者撑腰，充分调动广大干部群众想干事、敢干事、干成事的积极性和主动性。完善责任机制和督查机制，对为官不为、工作不力的严肃问责，对懒政怠政、失职渎职的严厉惩处，把求真务实的导向树起来，把真抓实干的规矩严起来，铆紧各方责任，层层传导压力，以务实推动发展，以实干赢得未来。

　　各位代表!未来五年承德发展的美好蓝图已经绘就，走好“加快转型、绿色发展、跨越提升”新路需要全市人民团结一心、众志成城、艰苦奋斗。让我们更加紧密地团结在以习近平同志为核心的党中央周围，在省委、省政府和中共承德市委正确领导下，开拓创新，拼搏进取，为实现脱贫摘帽、全面小康，建设生态强市、魅力承德而努力奋斗！
